# An Insight Into Adolescent Dermatitis Artefacta: A Case Report

**DOI:** 10.7759/cureus.68682

**Published:** 2024-09-05

**Authors:** Manavi Gupta, Apoorva Sharma, Muthu Sendhil Kumaran

**Affiliations:** 1 Department of Dermatology, Postgraduate Institute of Medical Education and Research, Chandigarh, IND

**Keywords:** adolescent, dermatitis artefacta, malingering, psychodermatology, psychosomatic

## Abstract

Dermatitis artefacta (DA) is a rare and challenging-to-diagnose factitious dermatological disorder, most commonly affecting late adolescents and young adults. This case report presents a 17-year-old girl with a history of unexplained linear lesions on her face and abdomen persisting for 11 months, leading to significant school absenteeism. The dermatological examination was otherwise unremarkable except for multiple well-defined excoriations, erosions, and scarring, suggestive of DA. Dermoscopic examination supported this diagnosis, showing characteristic features. The patient was treated with N-acetyl cysteine and referred for psychiatric evaluation, highlighting the intricate nature of managing DA, particularly in young individuals who may have underlying psychological distress. The case underscores the importance of a multidisciplinary approach in diagnosing and treating DA, given its overlap with other neuropsychiatric and dermatological disorders.

## Introduction

Dermatitis artefacta (DA) is a rare, challenging-to-diagnose factitious dermatological disorder, primarily affecting late adolescents to young adults [1]. It is three to five times more common in women than in males [2]. A retrospective study of 57 patients found that the prevalence was 2.8 times higher in females than in males and that 88% of patients had multiple lesions. All 57 patients were evaluated by a psychiatrist, and only 18% received a confirmed psychiatric diagnosis [3]. A diagnosis of DA should be considered when symptoms coexist in an unusual pattern or indicate several distinct conditions with no conclusive laboratory investigations.

## Case presentation

**Figure 1:**
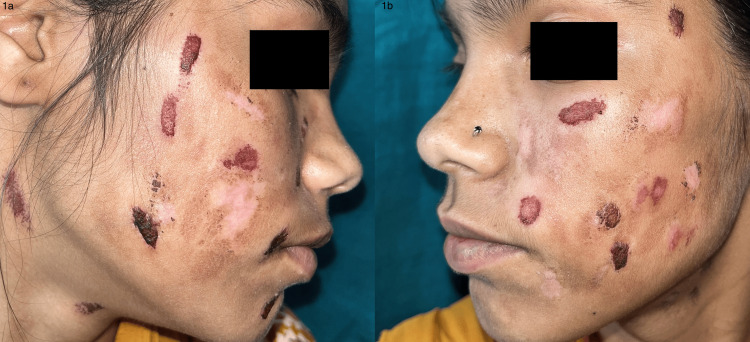
Seventeen-year-old female with multiple linear excoriations, erosions, and scarring on the face, involving predominantly bilateral malar areas

A 17-year-old girl presented with linear lesions on her face and abdomen for the past 11 months, causing nine months of school absenteeism. According to the patient, the lesions appeared suddenly, were asymptomatic, and improved within days, but left postinflammatory hypo- and hyperpigmentation and scarring. All hematological investigations at different hospitals came out normal. The patient exhibited indifference and an apathetic demeanor, with a notably incomplete history. Upon closer inspection, the face and abdomen showed numerous, linear, well-defined excoriations and erosions, along with crusting and scabs of various sizes and scarring (Figure 1).

**Figure 2:**
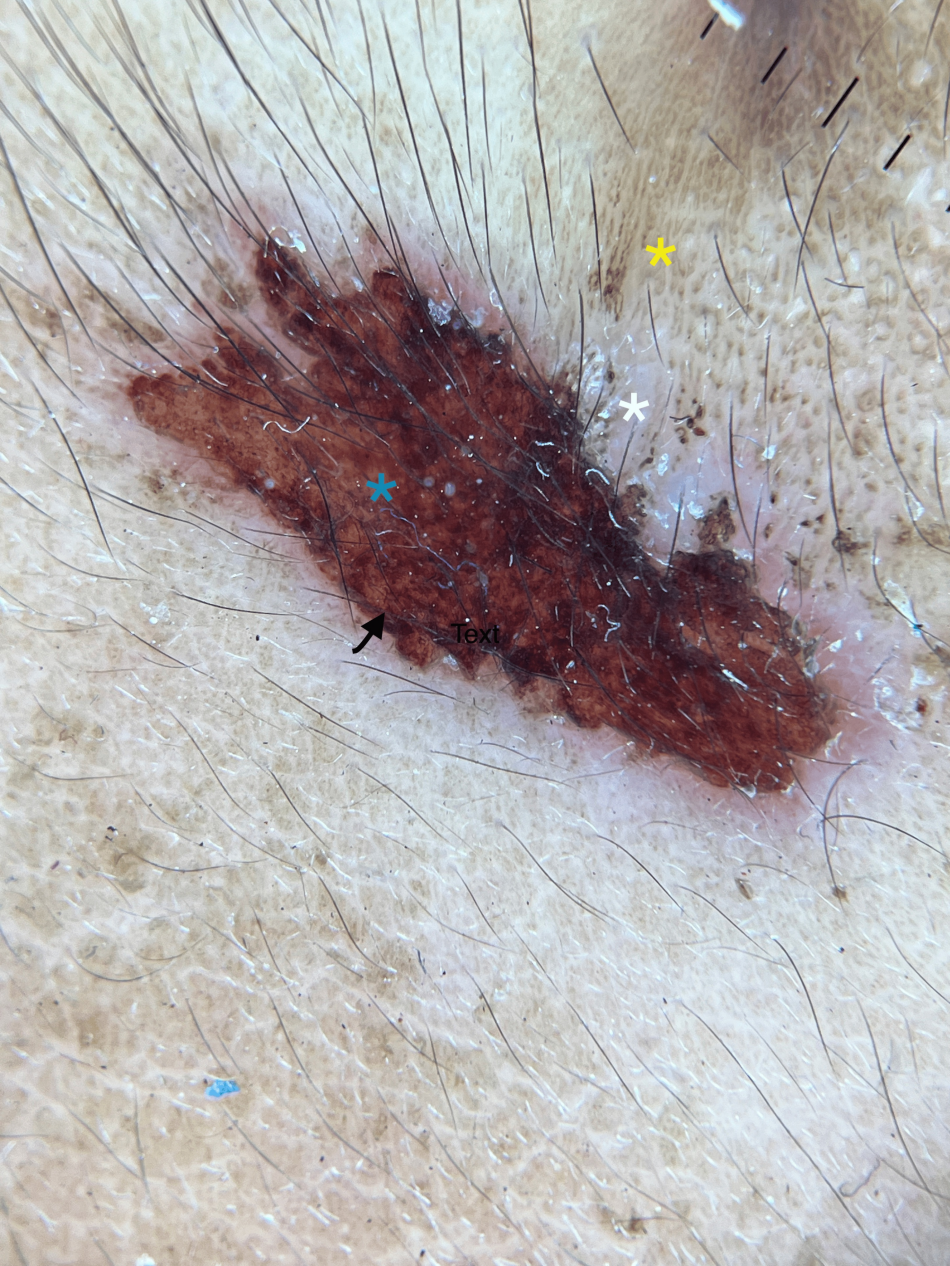
Dermoscopy reveals three distinct zones: an outer zone of hyperpigmentation (yellow star), a middle zone of hypopigmentation (white star), and central crusting (arrowhead). Red blotches are visible within the central crusting (blue star) (Dermlite DL4, Schuco, Nuremberg, Germany, polarized mode, ×10)

Dermoscopic examination revealed central crusting surrounded by a peripheral erythematous to whitish halo and an outer hyperpigmented area (Figure 2).

With a tentative diagnosis of DA based on the clinical symptoms and the previously mentioned history, the patient was started on N-acetyl cysteine 300 mg twice a day and was referred for psychiatric evaluation but could not be further followed up.

## Discussion

Managing DA is complex, especially with young women who may have personality disorders and deny psychological distress. Common clinical presentations in DA include excoriations, ulcers, and blisters, as seen in our case [4]. Apart from excoriations and erosions, ulcers are the second most prevalent presenting cutaneous symptom [5]. Dermoscopic findings that showed red blotches may indicate capillary leaking, and the yellowish crust may indicate serum exudation subsequent to excoriation, which was similar to a previously reported case [6]. They frequently have an odd shape and a sharp linear appearance [7], as seen in our case.

Other neuropsychiatric disorders like neurotic excoriation disorder involve scratching to relieve stress, while delusional parasitosis involves skin picking due to a false belief of bug infestation [8]. Neurotic excoriations involve repetitive scratching or picking driven by psychological issues like anxiety or obsessive-compulsive disorder, typically resulting in erosions or scabs on accessible areas. DA features a range of self-inflicted injuries, such as burning or gouging, often linked to emotional distress or a need for attention. DA lesions are usually more varied and may appear in geometric or irregular patterns. While both conditions involve self-injury, neurotic excoriations are primarily motivated by an urge to alleviate psychological discomfort, whereas DA reflects a broader range of intentional self-harm [9]. Our patient inflicted injuries on the most exposed part of her body, namely her face, and found psychiatric treatment difficult to accept and did not attend subsequent dermatological follow-ups favoring DA. Malingering involves faking symptoms for gain, while patients of Munchausen syndrome fabricate symptoms for attention.

Managing DA is complicated by the patient's and sometimes the family's resistance to psychiatric consultation, often because of the fear of stigmatization. This resistance can hinder effective treatment, as psychiatric evaluation and intervention are crucial components of managing DA.

## Conclusions

DA remains a challenging diagnosis due to its overlap with other psychocutaneous conditions and the deliberate nature of the self-inflicted lesions. This case highlights the importance of a thorough dermatological and psychological evaluation when faced with atypical, unexplained skin presentations, especially in young women. Early recognition and interdisciplinary management are crucial to prevent further physical and psychological deterioration, as well as to address any underlying psychiatric issues.
